# Groundwater radon exposure and risk of lung cancer: A population-based study in Finland

**DOI:** 10.3389/fonc.2022.935687

**Published:** 2022-09-15

**Authors:** Kishor Hadkhale, Janne Atosuo, Tuula Putus

**Affiliations:** Department of Clinical Medicine, Faculty of Medicine, University of Turku, Turku, Finland

**Keywords:** cancer, exposure, groundwater, lung, radon

## Abstract

Naturally occurring radioactive elements can be found in groundwater and exposure to such elements is associated with an increased risk of lung cancer. In this study, we aimed to observe the association between exposure to these radioactive elements in groundwater and the risk of lung cancer in selected regions in Finland. This is a population-based study from 1955 to 2019 in Finland. The exposed municipalities with their corresponding hospital districts were selected based on radon measurements at groundwater treatment plants. Lung cancer cases were obtained from the Finnish cancer registry. The 5-year incidence rates for lung cancer were calculated and a comparison was made between each of the hospital districts with radon exposure. More than 93,000 cases of lung cancer were reported in the radon-exposed regions over the examined period of 64 years. The highest number of cases was recorded in the Helsinki University hospital district and the least in the Southern Savo hospital district. Similarly, the lung cancer incidence rate was highest in Lapland and lowest in the Southern Savo hospital district. The number of daily smokers in the working-age population appears to have decreased in all the hospital districts from 2013 to 2018. A statistically significant increased risk of lung cancer was observed in the high radon-exposed hospital districts compared to those with lower exposure. Groundwater radon exposure is observed to be associated with an increased risk of lung cancer.

## Introduction

Radon exposure is a primary cause of lung cancer among non-smokers and the second leading cause among smokers worldwide (1). Epidemiological studies have reported that the synergistic effect of lung cancer risk is higher among smokers (both current and former smokers) exposed to radon (2–4). These studies reported that every 100 becquerels per cubic meter (Bq/m^3^) increase in radon is associated with an approximate 8% increase in the risk (95% CI 3.0–16.0) of lung cancer; this suggests an overall burden of a 10%–15% risk of lung cancer due to radon exposure (5). Pooled analysis of individual case–control studies from European and North American studies has shown a significantly increased risk of lung cancer due to residential radon exposure (6–9). A recent African study also showed an increased risk of cancer due to groundwater radon exposure (10). Findings from other Nigerian studies were consistent with exposure to radon in drinking water (11, 12). Naturally occurring radioactive elements are found in all groundwater and primarily in bedrock waters. Radon is the most significant factor among these elements as it dissolves in groundwater. Exposure to these radioactive elements increases the risk of cancer, and radon is primarily associated with the risk of lung cancer (13). Groundwater is a source of indoor air radon as radon transfers from the water to the air during various water-related activities such as laundry and during showers (14, 15). It is a colorless radioactive gas that forms naturally from the decay of radioactive elements. Radon in the soil can move freely in the air, underground water, and surface water. It can only be detected by radon analysis (4).

The overall incidents of lung cancer cases have increased in Finland since the early 1990s with the highest number of cases in the Helsinki University Hospital district and the lowest in the Oulu University Hospital district. However, the age-standardized incidence rate has decreased over the years and has been relatively stable since around the 2000s. In terms of gender, the age-standardized incidence rate has sharply decreased among men but slightly increased among women throughout the period (16). In Finland, according to STUK (the Radiation and nuclear safety authority), over 300 lung cancer cases per year are diagnosed due to radon alone. The International Agency for Research on Cancer (IARC) has classified radon as a group 1 carcinogenic for humans (2). Finland has a higher radon concentration compared to other European countries due to the geology, construction technology, climate, and uranium concentration in Finnish soil (17). The average radon concentration in Finnish homes is approximately 94 Bq/m^3^ (18). However, there is a quite large regional difference. In Finland, STUK is responsible for safety, measurement, monitoring, control, security, and development regulations for all types of radiation. Systematic surveys of radon concentration in Finnish groundwater were already started in the 1960s. However, the complete information for radon concentration is still unclear. With the new radiation act (2018) in place, it is expected that better expertise in terms of radon measurements will be possible throughout the country and primarily in high-risk municipalities. This study aims to observe the association between groundwater radon exposure and the risk of lung cancer.

## Materials and methods

This is a population-based study in Finland. The exposed municipalities were selected based on STUK’s indoor radon measurements at groundwater treatment plants (19). According to the radiation act (859/2018), authorities are obliged to measure radon in the indoor air at water treatment plants if the indoor air at the workplace is in contact with groundwater or artificial groundwater.

A total of 425 radon measurements at both groundwater and artificial groundwater facilities were made in 2020. The radon measurements were carried out in 211 workplaces by 51 employers. The study was conducted in 59 municipalities corresponding to the14 hospital districts throughout Finland (**Table 1**). Details about exposure in the municipalities are available in the following (19). Municipalities with radon exposure are categorized based on exposure estimates. These are categorized as follows: a maximum exposure value of <334 Bq/m^3^ (no exposure), 334–1,499 Bq/m^3^ (low exposure) 1,500–10,500 Bq/m^3^ (medium exposure), and >10,500 Bq/m^3^ (high exposure). However, for our analysis, we categorized no exposure, low exposure, and medium exposure as low-risk hospital districts and high exposure as high-risk hospital districts. Likewise, lung cancer cases were calculated from the Finnish Cancer Registry (https://cancerregistry.fi/statistics/cancer-statistics/). In this study, we calculated the cancer cases from 14 selected hospital districts corresponding to their respective radon-exposed municipalities. A unique personal identification number provided to all individuals in the Finnish population helped in obtaining various information about health and other matters. The Finnish Cancer Registry obtains cancer cases using this personal identification number from the population registry. The exact 95% confidence intervals (CIs) of the incidence rates were defined based on Poisson regression. We used two-sided tests to obtain a statistical significance level of 0.05. We were unable to identify the incident cancer cases in each of the exposed municipalities due to the smaller number of cases. Hence, according to the data security and ethical guidelines, we estimated the lung cancer cases based on the hospital districts in the respective municipalities. There are altogether 22 hospital districts within the five university hospital districts in Finland.

**Table 1:** Concentrations of indoor air radon at groundwater treatment plants in Finnish municipalities in 2020.

	Municipalities	Min.	Max.	Above reference level (%)	Hospital districts	University hospital districts
1	Eura	39	693	50	Satakunta	TYKS
2	Forssa	14	376	17	Kanta-Häme	TAYS
3	Haapajärvi	3930	3,930	100	Northern Ostrobothnia	OYS
4	Hanko	7	7,384	23	Uusimaa	HYKS
5	Harjavalta	45	701	25	Satakunta	TYKS
6	Hattula	33	2,630	50	Kanta-Häme	TAYS
7	Hausjärvi	448	1,780	100	Kanta-Häme	TAYS
8	Hollola	120	5,340	65	Päijät-Häme	HYKS
9	Honkajoki	184	465	50	Satakunta	TYKS
10	Huittinen	52	160	0	Satakunta	TYKS
11	Hyvinkää	28	1,100	25	Uusimaa	HYKS
12	Hämeenlinna	15	3,620	69	Kanta-Häme	TAYS
13	Iitti	6720	6,720	100	Päijät-Häme	HYKS
14	Imatra	68	1,267	67	South Karelia	HYKS
15	Janakkala	33	9,928	29	Päijät-Häme	TAYS
16	Joensuu	41	21,257	64	North Karelia	KYS
17	Joutsa	192	1,697	67	Central Finland	KYS
18	Jyväskylä	131	1,572	50	Central Finland	KYS
19	Kajaani	4	847	20	Kainuu	OYS
20	Kalajoki	289	1,686	80	Northern Ostrobothnia	OYS
21	Kangasala	64	8,026	72	Tampere region	TAYS
22	Kankaanpää	358	399	100	Satakunta	TYKS
23	Karvia	1567	1,567	100	Satakunta	TYKS
24	Kitee	2	1,312	29	North Karelia	KYS
25	Kontiolahti	124	14,935	80	North Karelia	KYS
26	Kouvola	16	6,370	62	Kymenlaakso	HYKS
27	Kuopio	5	4,740	27	Northern Savo	KYS
28	Lahti	10	2,100	69	Päijät-Häme	HYKS
29	Lappeenranta	60	6,260	73	South Karelia	HYKS
30	Laukaa	35	6,451	43	Central Finland	KYS
31	Leppävirta	180	3,530	86	Northern Savo	KYS
32	Miehikkälä	294	294	0	Kymenlaakso	HYKS
33	Mäntsälä	17	348	20	Uusimaa	HYKS
34	Mäntyharju	2101	4,976	100	Southern Savo	KYS
35	Nakkila	95	95	0	Satakunta	TYKS
36	Nokia	95	540	50	Tampere region	TAYS
37	Nurmes	2253	2,955	100	North Karelia	KYS
38	Nurmijärvi	290	1,120	50	Uusimaa	HYKS
39	Orimattila	1663	6,130	100	Päijät-Häme	HYKS
40	Orivesi	765	1,071	100	Tampere region	TAYS
41	Outokumpu	355	1,186	100	North Karelia	KYS
42	Parikkala	111	582	50	South Karelia	HYKS
43	Pori	8	10	0	Satakunta	TYKS
44	Pornainen	221	613	50	Uusimaa	HYKS
45	Pukkila	110	202	0	Päijät-Häme	HYKS
46	Riihimäki	39	440	33	Kanta-Häme	TAYS
47	Rovaniemi	880	31,600	100	Lapland	OYS
48	Sievi	780	10,610	100	Northern Ostrobothnia	OYS
49	Siikainen	69	266	0	Satakunta	TYKS
50	Siilinjärvi	136	3,013	71	Northern Savo	KYS
51	Sipoo	5690	5,690	100	Uusimaa	HYKS
52	Säkylä	212	237	0	Satakunta	TYKS
53	Tampere	27	10,360	72	Tampere region	TAYS
54	Tohmajärvi	20	8,964	50	North Karelia	KYS
55	Tuusniemi	10420	10,550	100	Northern Savo	KYS
56	Tuusula	80	530	33	Uusimaa	HYKS
57	Ulvila	129	1,935	67	Satakunta	TYKS
58	Valtimo	530	530	100	North Karelia	KYS
59	Ylöjärvi	140	14130	63	Tampere region	TAYS

Source: STUK report 2020.

The results are presented based on cancer incidences in each of the selected hospital districts from 1955 to 2019. Lung cancer cases were estimated according to the 5-year incidence rate and stratified by gender. Incidence rates are presented both in an overall rate of per 100,000 and in the age-standardized rate of Finland (2014) (**Table 2**). Similarly, the high- and low-risk municipalities were categorized based on the quantitative radon exposure estimates at the selected groundwater treatment plants (19). This study includes all the lung cancer cases from all of the exposed municipalities. The five selected university hospital districts included the Helsinki University Hospital district (HYKS) which is composed of the Uusimaa hospital district (without the Helsinki region), the Kymenlaakso hospital region, the Päijät-Häme hospital district, and the South Karelia hospital district. The Turku University Hospital district (TYKS) includes the Satakunta hospital district. Similarly, the Tampere University Hospital district (TAYS) consists of the Kanta-Häme and Pirkanmaa hospital districts. The Kuopio University Hospital district (KYS) is composed of the Southern Savo hospital district, the North Karelia hospital district, the Northern Savo hospital district, and the central Finland hospital district. The Oulu University Hospital district (OYS) includes the Northern Ostrobothnia hospital district, Kainuu hospital district, and Lapland hospital district (**Table 3**).

**Table 2:** The incidence rate and total number of diagnosed lung cancer cases in Finland from 1955 to 2019, by hospital district.

	Hospital districts	^*^Incidence rate	^**^ASR (FIN)	Diagnosed cases
1	Central Finland hospital district	503.5	722.0	5,854
2	Kainuu hospital district	601.1	862.6	2,649
3	Kanta-Häme hospital district	584.5	732.3	4,716
4	Kymenlaakso hospital district	591.3	737.5	5,395
5	Lapland hospital district	603.0	932.1	3,751
6	North Karelia hospital district	655.9	885.3	6,033
7	Northern Ostrobothnia hospital district	516.6	853.4	9,014
8	Northern Savo hospital district	580.2	796.1	7,431
9	Päijät-Häme hospital district	543.4	709.0	5,483
10	Satakunta hospital district	590.2	758.9	6,880
11	South Karelia hospital district	589.0	732.2	4,094
12	Southern Savo hospital district	579.6	694.8	3,412
13	Tampere region hospital district	541.1	714.4	12,372
14	Uusimaa without Helsinki	480.0	811.7	16,606

*The number of new cancer cases per 100,000 people per year.

**The number of new cancer cases per 100,000 people per year, if the age structure of the population remains similar to that in Finland in 2014.

**Table 3:** Radon exposure information in the selected municipalities and hospital districts in Finland.

SN	Hospital districts	Municipalities	University hospital districts
1	Uusimaa hospital district (without Helsinki)	Hyvinkää, Mäntsälä, Nurmijärvi, Pornainen, Tuusula, Hanko, Sipoo	Helsinki University Hospital district.
2	Kymenlaakso hospital district	Miehikkälä, Kouvola	Helsinki University Hospital district.
3	Päijät-Häme hospital district	Pukkila, Hollola, Iitti, Lahti, Orimattila	Helsinki University Hospital district.
4	South Karelia hospital district	Imatra, Parikkala, Lappeenranta	Helsinki University Hospital district
5	North Karelia hospital district	Joensuu, Kitee, Kontiolahti, Nurmes, Outokumpu, Tohmajärvi, Valtimo	Kuopio University Hospital district
6	Northern Savo hospital district	Kuopio, Leppävirta, Siilinjärvi, Tuusniemi	Kuopio University Hospital district
7	Southern Savo hospital district	Mäntyharju	Kuopio University Hospital district
8	Central Finland hospital district	Joutsa, Jyväskylä, Laukaa	Kuopio University Hospital district
9	Northern Ostrobothnia hospital district	Haapajärvi, Kalajoki, Sievi	Oulu University Hospital district
10	Lapland hospital district	Rovaniemi	Oulu University Hospital district
11	Tampere region (Pirkanmaa) hospital district.	Kangasala, Nokia, Orivesi, Tampere, Ylöjärvi	Tampere University Hospital district
12	Kanta-Häme hospital district	Riihimäki, Forssa, Hattula, Hausjärvi, Hämeenlinna, Janakkala	Tampere University Hospital district
13	Kainuu hospital district	Kajaani	Oulu University Hospital district
14	Satakunta hospital district	Honkajoki, Huittinen, Nakkila, Pori, Siikainen, Säkylä, Eura, Harjavalta, Kankaanpää, Karvia, Ulvila	Turku University Hospital district

## Results

**Figure 1:**
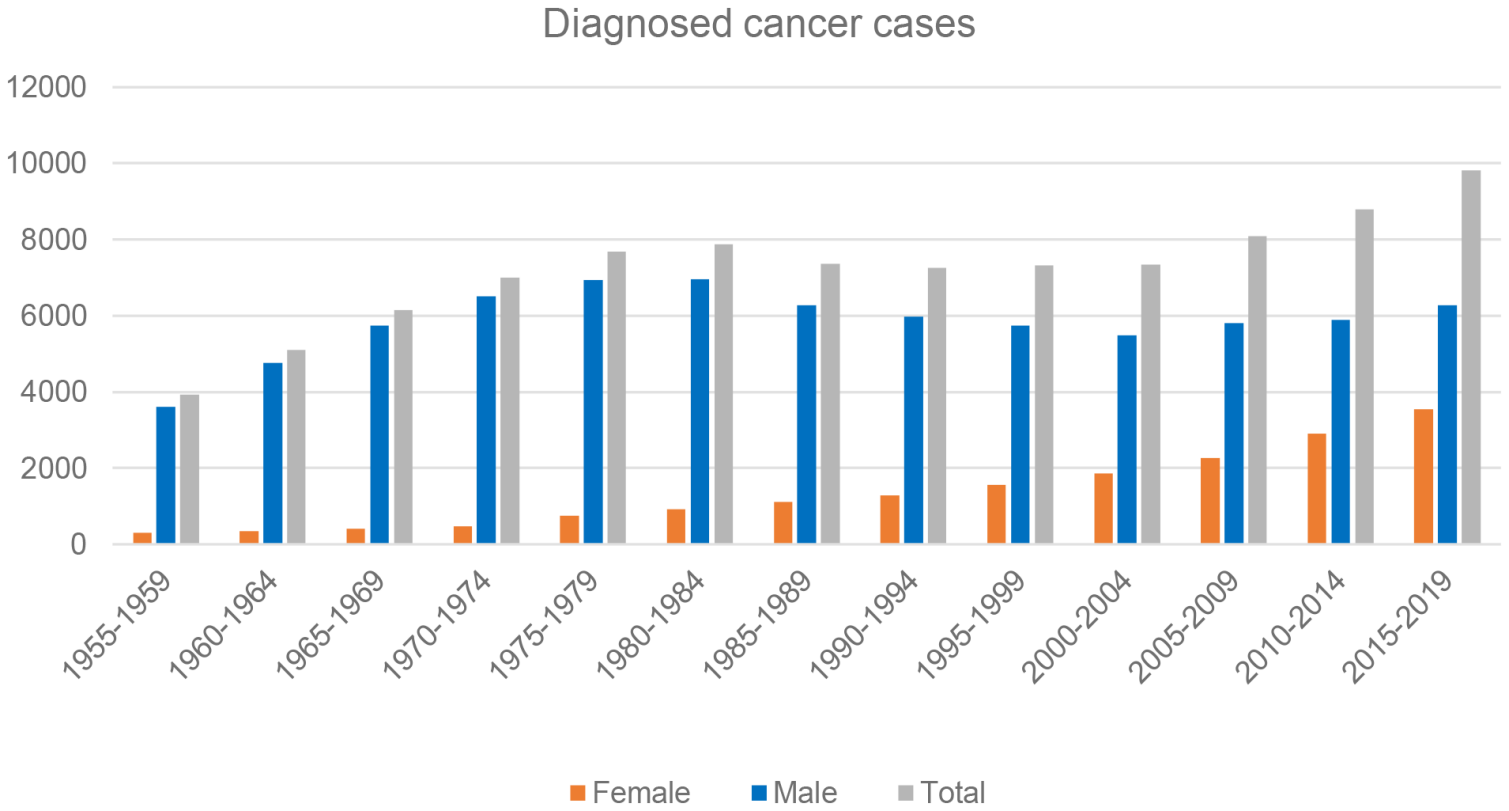
Diagnosed lung cancer cases by gender in selected radon-exposed municipalities in Finland from 1955 to 2019.

**Figure 2:**
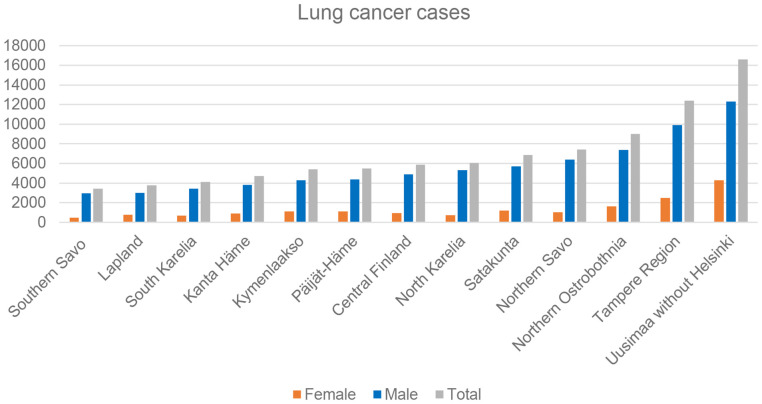
Diagnosed lung cancer cases by hospital district in Finland from 1955 to 2019.

**Figure 3:**
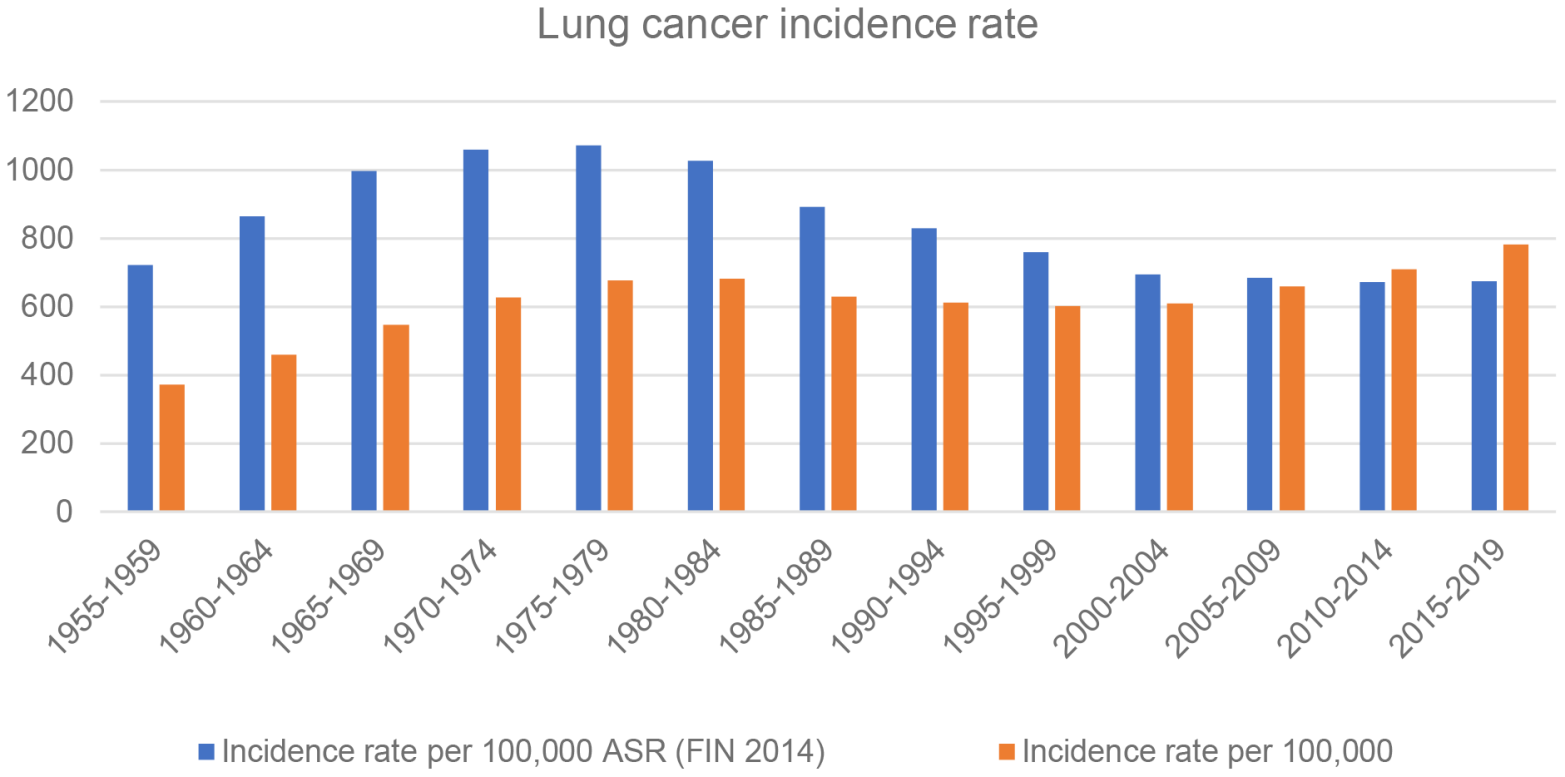
Lung cancer incidence rates in radon-exposed municipalities in Finland from 1955 to 2019. The number of new cancer cases per 100,000 people per year, if the age structure of the population remains similar to that in Finland in 2014. The number of new cancer cases per 100,000 people per year.

More than 93,000 cases of lung cancer cases were reported by the radon-exposed hospital districts from 1955 to 2019. The majority of the cases (approximately 80%) were men (**Figure 1**). The highest number of lung cancer cases were diagnosed in the Uusimaa hospital district (n = 16,606) and Pirkanmaa hospital district (n = 12,372), whereas the least were from the Southern Savo hospital district (n = 3,412) and Lapland hospital district (n = 3,751 (**Figure 2**). During this period, the incidence rate of lung cancer increased until 1985 and then started to decrease throughout this region until late 1990. However, the incidence again started to increase after 2000 and has continued to increase (**Figure 3**).

**Figure 4:**
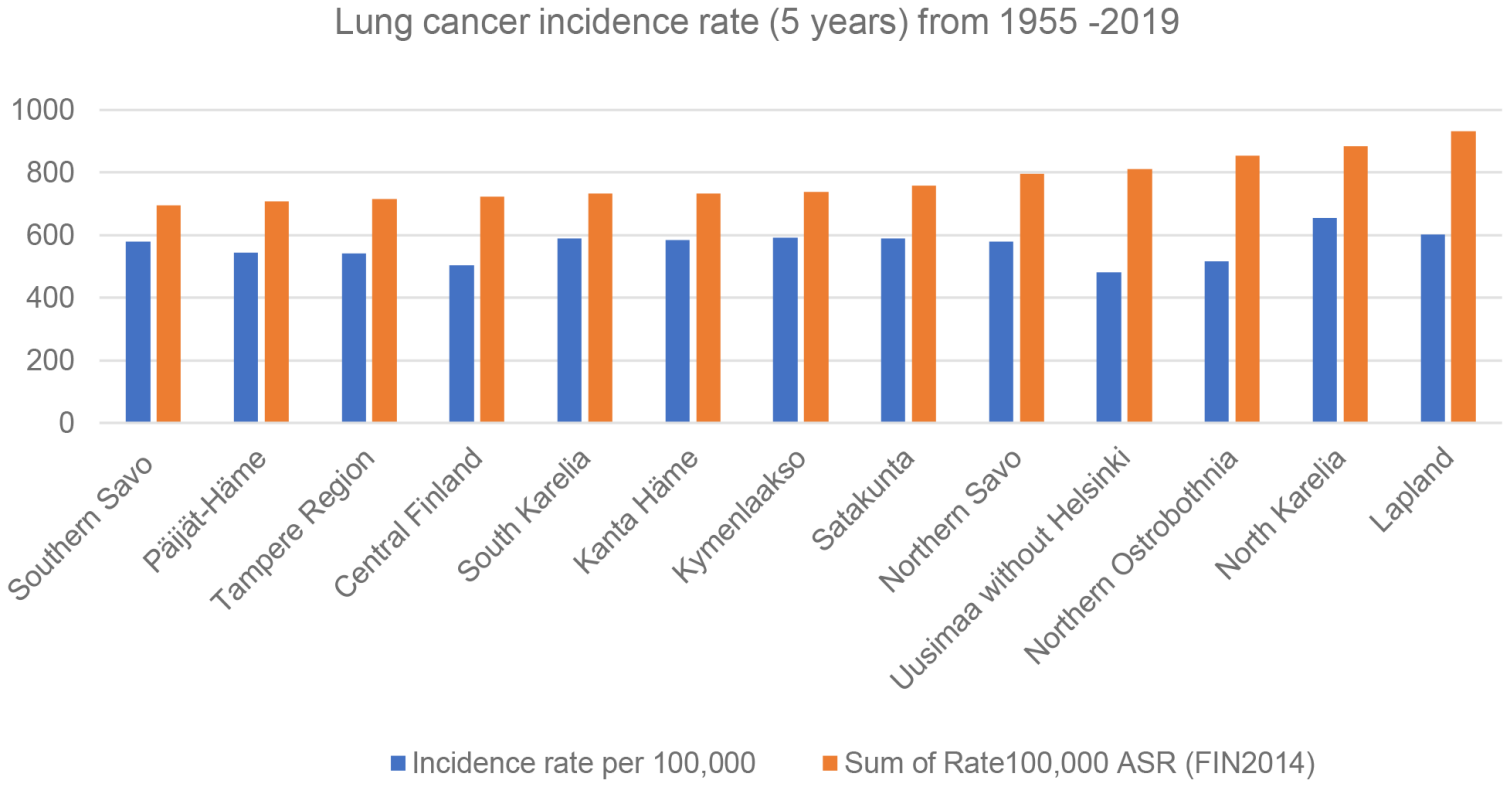
Lung cancer incidence rates in radon-exposed municipalities with their corresponding hospital districts in Finland from 1955 to 2019. The number of new cancer cases per 100,000 people per year. The number of new cancer cases per 100,000 people per year, if the age structure of the population remains similar to that in Finland in 2014.

**Figure 5:**
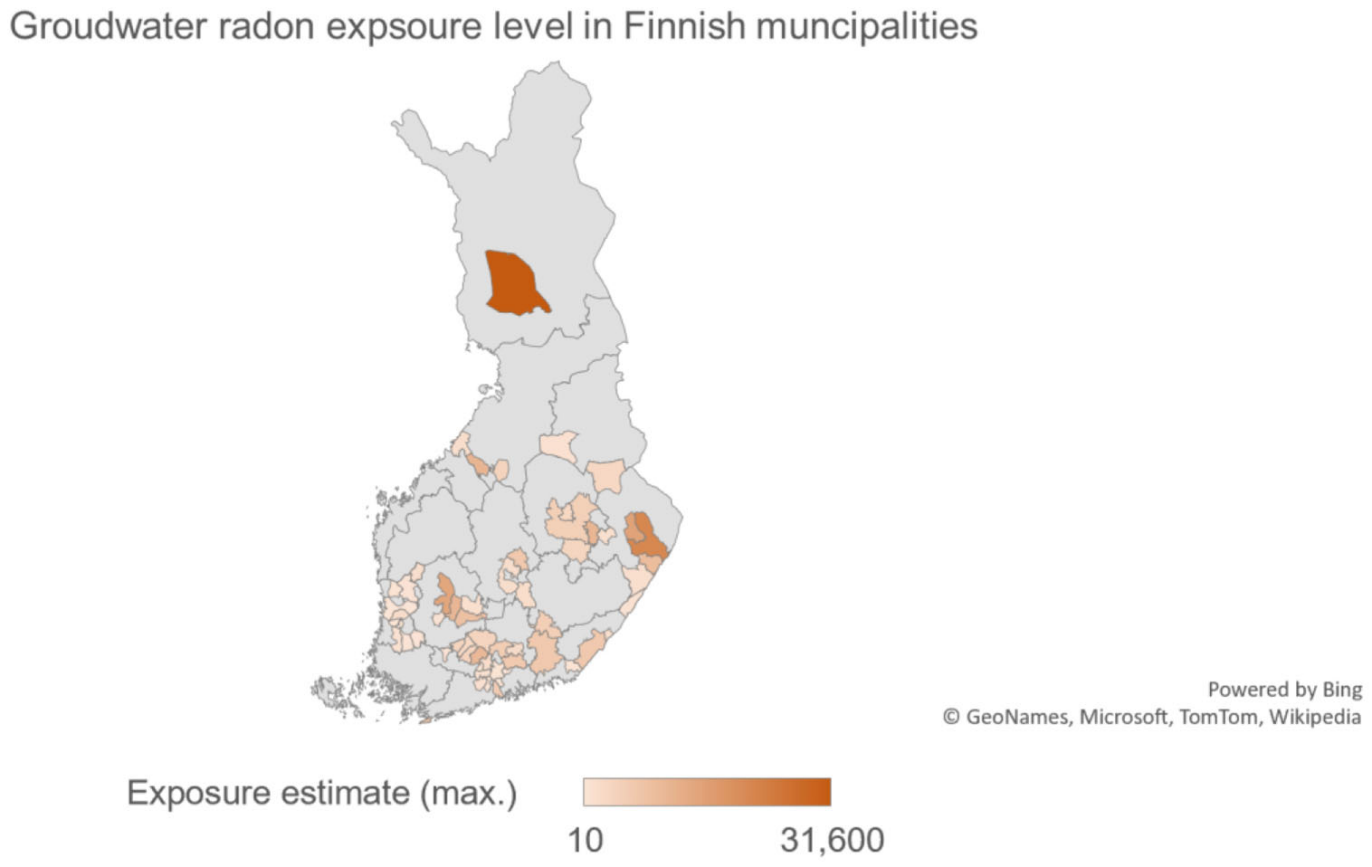
Map of Finland indicating radon exposure areas. Source: Senja et al., 2021.

In the stratification based on the hospital districts, it was observed that the Lapland, North Karelia, and Northern Ostrobothnia hospital districts had the highest incidence rates whereas Southern Savo, central Finland, Päijät-Häme, the Tampere region (Pirkanmaa), and South Karelia had lower incidence rates. The age-standardized incidence rate was highest in Lapland (932.05), North Karelia (885.25), and Northern Ostrobothnia (853.42) and lowest in the Southern Savo and Päijät-Häme hospital districts according to the age structure of the Finnish population in 2014 (**Figure 4**). Accordingly, the radon exposure estimate was observed to be the highest in the Lapland, North Karelia, and Northern Ostrobothnia hospital districts. The highest mean average risk (>10,000 Bq/m^3^) was observed in Rovaniemi (Lapland hospital district), Joensuu and Kontiolahti (North Karelia Hospital district), Ylöjärvi (Pirkanmaa hospital district), Sievi (Northern Ostrobothnia hospital district), and Tuusniemi (Northern Savo hospital district). Similarly, low to no risk was observed in the following municipalities: Huittinen, Nakkila, Pori, Siikainen, Säkylä, Eura, Harjavalta, Honkajoki, Kankaanpää (Satakunta hospital district) Pukkila (Päijät-Häme hospital district), Miehikkälä (Kymenlaakso hospital district), Orivesi, Nokia (Pirkanmaa hospital district), Riihimäki, Forssa (Kanta-Häme hospital district), and Imatra and Parikkala (South Karelia hospital districts) (**Tables 1**, **3** and **Figure 5**). A statistically significant increased risk was observed in the high-risk hospital districts as compared to the low-risk hospital districts. In comparison with the reference category, the highest risks were observed at 1.18, 1.14–1.23; 1.17, 1.13–1.22; and 1.13, 1.08–1.17 in Lapland, North Karelia, and Northern Ostrobothnia hospital districts, respectively (**Table 4**).

**Table 4:** Lung cancer incidence rates in Finland from 1955 to 2019 in radon-exposed municipalities with their corresponding hospital districts.

Hospital districts	University hospitals	IRR	95% CI
Reference (low)	Others	1.00	Reference
Lapland hospital district	Oulu University Hospital district	1.18	1.14-1.23
North Karelia hospital district	Kuopio University Hospital district	1.17	1.13-1.22
Northern Ostrobothnia	Oulu University Hospital district	1.13	1.08-1.17

Low-risk hospital districts (reference): Central Finland hospital district, Kanta-Häme hospital district, Kymenlaakso hospital district, Northern Savo hospital district, Päijät-Häme hospital district, Satakunta hospital district, South Karelia hospital district, Southern Savo hospital district, The Tampere region hospital district, Uusimaa not including the Helsinki hospital region.

High-risk hospital districts: Lapland hospital district, North Karelia hospital district, and Northern Ostrobothnia hospital district.

## Discussion

Based on the radon concentration at the groundwater treatment plants and the incidences of lung cancer, our study observed that almost all the municipalities with their corresponding hospital districts with the highest radon exposure level correlated with a higher incidence rate of lung cancer as compared to municipalities with lower or no exposure levels. Hence, we can conclude that groundwater radon exposure is associated with the increased risk of lung cancer in these regions.

Studies on indoor radon exposures and lung cancer are well represented by prior research, including the pathway combination for radon (6, 9, 20), whereas there are very few studies on the association between groundwater radon and the risk of cancer. Our findings were consistent with one other epidemiological study on groundwater radon concentration and lung cancer risk. This study, conducted in North Carolina, USA, observed a significant positive association between groundwater radon concentration and lung cancer incidence rates (20). According to the study, groundwater radon exposure was associated with an odds ratio (OR) of 1.13 (95% CI 1.04–1.23), suggesting an overall 3% increase in lung cancer incidence rate for every 100-Bq/l increase. The study was adjusted for various confounding factors such as age, gender, smoking, race, and indoor air radon, and the incidence of cancer cases was obtained from the North Carolina Central Cancer Registry. Another study conducted in Maine observed a similar positive significant correlation with lung cancer (21). The study measured the groundwater radon concentration from 16 different counties. According to the European Environment and Health Information System report, Finland belongs to one of the European countries more prone to radon emissions (17). In Finland, systematic surveys of groundwater radon exposure were already started in the 1960s. The survey was initiated in the Helsinki area where a mean exposure of 1,600 Bq/l was observed (22). This worrying figure obliged the authorities to survey waterborne radon throughout Finland. After the 1970s, the survey mainly focused on private wells and evaluating new groundwater sources before connection to local distribution networks (13). According to the most recent legislation, when the exposure of workers to radon is higher than the reference value, the workplace is placed under radon control and the workers are monitored until the exposure value among the workers is reduced below the threshold reference value. After this period of improvement, mitigation measures must be applied to limit the exposure within a certain deadline. For water utilities, the deadline is usually 1 year and the actions have to be measured and verified. For example, when the employee’s working hours exceed 600 at the same workplace, a reference value of 300 Bq/m^3^ must be observed. The annual average is estimated during the measurement period (value 0.9). This reference value of 300 Bq/m³ thus corresponds to a measurement result of 334 Bq/m³ (19). Every employer is responsible for notifying the authorities of the radon measurements that occur at the workplace. In this study, we have excluded the Helsinki municipality because Helsinki receives raw water from Lake Päijänne *via* a tunnel. The strengths of the study include the long follow-up time, the high reliability and validity of the exposure data provided by STUK, and smoking information from the National Institute of Health and Welfare (THL) and the Finnish Cancer Registry; this information made it possible to avoid biases such as the recall bias in the dataset.

**Figure 6:**
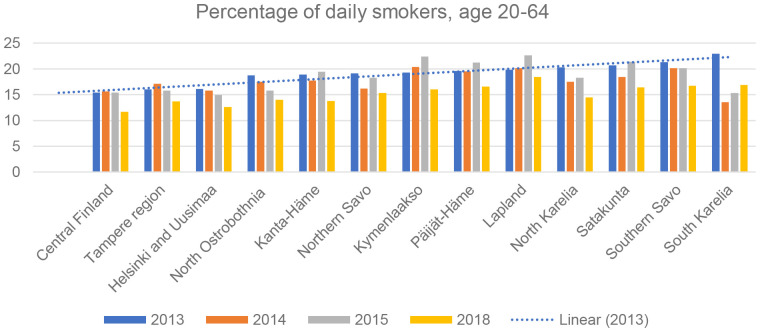
Total number of daily smokers in different hospital districts from 2013 to 2018 in Finland.

The limitations of the study include the usual ecological study design where the incidence rate is assigned at the population level. In this study, we were not able to adjust for possible confounding factors such as smoking despite strong evidence of smoking and lung cancer risk. Similarly, the cancer cases were not available at the municipal level but rather at the hospital district level. This could weaken the association between the exposure variables and the outcome of interest. However, we were able to identify an association between rare exposures that had not been widely studied. Hence, this could initiate further research on individual-level exposure estimates and a smoking-adjusted risk of lung cancer. The purpose of this study was also to investigate the current situation of lung cancer cases in highly exposed regions and monitor the health of the population. Furthermore, the study could help develop and strengthen the legislation on radon exposure associated with public health risks. Since the incidence of radon-exposed lung cancer is very low, smoking adjustment could be difficult because the outcome is very rare. However, we have a significant sample size obtained from the Finnish Cancer Registry for the available exposure data. A Finnish database concerning smoking shows that the number of daily smokers, both men and women aged 20–64 years, has decreased in recent years in Finland (23). According to the database, in 2013, the highest number of daily smokers comprised residents in the South Karelia hospital district (22.9%) and the lowest in central Finland (15.4%) (**Figure 6**). The same data also show that some reference hospital districts in our study observed a higher number of daily smokers in specific years. However, information was limited to the years 2013–2015 and 2018 and we cannot make any conclusions without more detailed information. The completeness and accuracy of the cancer registers are considered to be of high quality in international rankings (24). However, we were unable to find the association between radon exposure and lung cancer risk in the Pirkanmaa hospital region where the groundwater radon exposure estimates are relatively higher. Geographically, Tampere is situated between two large lakes. The Tampere region uses considerable amounts of surface water which could be one reason why the cancer cases, mainly lung cancer cases, are not in line with the higher level of groundwater radon exposure compared to the northern municipalities (25). In our study, we were not able to identify any chemical compounds other than radon as being present in groundwater that could lead to the risk of cancers including lung cancer. However, the effect would be very low or not correlated. One of the earlier studies on groundwater in Northern Finland showed that other groundwater components such as iron, nitrates, and uranium were negatively correlated (26). In this study, a strong positive correlation was observed mainly for overall cancer and lung cancer in both men and women. Hence, this could be the indication of the risk of radon exposure for lung cancer. Despite this, we were not able to identify a cause–effect relationship in this study.

## Conclusion

Based on these findings, we were able to observe an association between groundwater radon exposure and the risk of lung cancer. Future studies with high-quality individual-level quantitative exposures will be required to explore the association between lung cancer and possibly other cancer risks due to radon exposure.

## Data availability statement

Publicly available datasets were analyzed in this study. This data can be found here: https://cancerregistry.fi/statistics/cancer-statistics/.

## Author contributions

KH, JA, and TP designed, analyzed and prepared the manuscript. KH, JA, and TP revised, reviewed, provided critical feedback and approved the manuscript for final submission.

## Conflict of interest

The authors declare that the research was conducted in the absence of any commercial or financial relationships that could be construed as a potential conflict of interest.

## Publisher’s note

All claims expressed in this article are solely those of the authors and do not necessarily represent those of their affiliated organizations, or those of the publisher, the editors and the reviewers. Any product that may be evaluated in this article, or claim that may be made by its manufacturer, is not guaranteed or endorsed by the publisher.
